# Generation, characterization, and application of caprine herpesvirus 1 secreted glycoprotein D

**DOI:** 10.1128/spectrum.02373-25

**Published:** 2025-11-28

**Authors:** Sergio Minesso, Amienwanlen Eugene Odigie, Valentina Franceschi, Simone Taddei, Vittorio Madia, Maria Tempesta, Gaetano Donofrio

**Affiliations:** 1 Department of Veterinary Science, University of Parma 9370, Parma, Italy; 2 Department of Veterinary Medicine, University of Bari 9295, Valenzano, Italy; Penn State College of Medicine, Hershey, Pennsylvania, USA

**Keywords:** experimental infection, ELISA, animal model for HSV2, caprine herpesvirus 1

## Abstract

Caprine herpesvirus 1 (CpHV-1), a member of the *Herpesvirales* order, *Herpesviridae* family, *Alphaherpesvirinae* subfamily, and *Simplexvirus* genus, is classically associated with two distinct clinical syndromes. In kids, CpHV-1 induces severe systemic disease with high morbidity and mortality, whereas in adult goats, the infection leads to genital lesions such as vulvovaginitis or balanoposthitis, with abortions occurring mainly in the second half of gestation. CpHV-1 shares several biological characteristics with human herpesvirus 2, including molecular features, tropism for vaginal epithelium, genital lesion nature, and latency in the sacral ganglia. These features make CpHV-1-infected goats a reliable animal model for studying human herpesvirus-induced genital disease, relevant for pathogenic research, as well as the development of new vaccines and antiviral agents. Recent full sequencing of the CpHV-1 genome has identified at least 10 genes encoding glycoproteins. Among these, glycoprotein D (gD) has been characterized but not yet exploited for immunogenic or diagnostic purposes. In this study, the structural features of CpHV-1 gD were predicted using *in silico* analysis. A truncated version of gD lacking the transmembrane domain (secreted glycoprotein D [Sec-gD]) was subsequently generated and expressed in mammalian cells, enabling its secretion into the culture medium. Despite the structural modifications, Sec-gD retained a conserved glycosylation pattern, as confirmed by N-glycosylation mutants generation and peptide-N-glycosidase F treatment. Furthermore, the antigenic properties of Sec-gD were preserved, as demonstrated by reverse serum neutralization assays. Notably, the culture supernatant containing Sec-gD was directly usable in diagnostic enzyme-linked immunosorbent assays, supporting its potential as a valuable tool for both diagnostic and immunization strategies.

**IMPORTANCE:**

Caprine herpesvirus 1 (CpHV-1)-infected goats represent a large animal model for studying human herpesvirus-induced genital disease and could be utilized for pathogenic research, as well as for the development of new vaccines and antiviral agents. CpHV-1 glycoprotein D can be efficiently produced and rescued from the supernatant of transfected mammalian cells, retaining its immunogenic properties, and could be used for immunogenic and diagnostic purposes.

## INTRODUCTION

Caprine herpesvirus 1 (CpHV-1) is a member of the *Herpesvirales* order, within the *Herpesviridae* family, *Alphaherpesvirinae* subfamily, and *Simplexvirus* genus (1). This virus is associated with two distinct clinical syndromes in goats: a fatal systemic illness in kids (2) and a genital disease in adults, which can manifest as balanoposthitis (3), vulvovaginitis (4), and abortion (5). Although only one complete genome sequence of CpHV-1 has been determined so far (https://www.ncbi.nlm.nih.gov/nuccore/NC_076509; accession number: NC_076509), restriction site maps have been developed using double digestion and cross-hybridization of individual restriction fragments. The molecular weight of CpHV-1 DNA, estimated by summing the weights of fragments generated by various endonucleases, is approximately 137 kbp (6, 7). From a pathogenic perspective, CpHV-1 infection typically begins at the respiratory or genital mucosa. The virus then disseminates systemically via a mononuclear cell-associated viremia, potentially leading to abortion in pregnant animals. CpHV-1 is shed through ocular, nasal, and genital secretions, with the genital tract considered the primary site for viral entry and persistence within herds (4). In kids, CpHV-1 causes a severe systemic disease marked by high morbidity and mortality, with ulcerative and necrotic lesions throughout the gastrointestinal tract. In adult goats, the infection results in genital lesions such as vulvovaginitis or balanoposthitis. Abortions typically occur in the second half of gestation and can be experimentally induced through intranasal or intravenous inoculation of pregnant goats (8). Following intravaginal infection, the virus establishes latency in the sacral ganglia. Reactivation may occur under physiological stress, particularly during the breeding season, and may be influenced by hormonal changes during estrus. However, experimental reactivation is challenging and generally requires high doses of dexamethasone (9). Notably, CpHV-1 shares several biological characteristics with human herpesvirus 2 (HSV-2) and bovine herpesvirus 1 (BoHV-1), including molecular features, tropism for vaginal epithelium, the nature of genital lesions, and latency in the sacral ganglia (10). CpHV-1 genomes have been recently fully sequenced (https://www.ncbi.nlm.nih.gov/nuccore/NC_076509; accession number: NC_076509) and exhibit the characteristic structure of a class D herpesvirus genome. This genome class is composed of a linear double-stranded DNA molecule, organized into a unique long segment and a unique short (US) segment. The US segment is flanked by internal and terminal inverted repeat sequences (IR and TR, respectively). The complete genome sequence of CpHV-1 has revealed the presence of at least 10 genes encoding glycoproteins. These glycoproteins have been identified and named based on their homology with those of herpes simplex virus type 1 (HSV-1) (11). Among these glycoproteins, glycoprotein D has been characterized (12); however, it has not been exploited as an immunogen or in terms of diagnostics. In this work, we successfully expressed and characterized glycoprotein D (gD) in a secreted form (Sec-gD) in the medium of a mammalian cell, and the medium containing Sec-gD could be directly employed for the development of a diagnostic enzyme-linked immunosorbent assay or immunization purposes.

## MATERIALS AND METHODS

### *In silico* analysis of CpHV-1 glycoprotein D

The amino acid sequence of CpHV-1 gD was retrieved from NCBI (https://www.ncbi.nlm.nih.gov/protein/; accession number: AAZ66865.1) in FASTA format and used as input for the downstream analyses. PSIPRED 4.0 (13) and GOR IV web server (14) were employed for secondary structure prediction. Prediction of transmembrane helices and signal peptide was performed with Phobius web server (15), and visualization of protein topology was carried out with Protter version. 1.0 (16). Prediction of glycosylation sites was performed with NetGlyc 1.0 (17). Template-based 3D modeling of CpHV-1 gD was performed using the SWISS-MODEL web server (18). A total of 172 templates matched the CpHV-1 gD sequence. Due to the unavailability of CpHV-1 gD templates, the prediction was based on crystal structures of the glycoprotein D from related herpesviruses (File S3). From these, the 10 most suitable templates were selected for further modeling. The resulting best-fitting model was selected based on sequence coverage, identity, Global Model Quality Estimate (GMQE), and the QMEANDisCo Global value. GalaxyRefine (19) was then employed to enhance the prediction accuracy. Both crude and refined models were evaluated with the SWISS-Model structure assessment tool. The final model was deposited in ModelArchive (Model ID: ma-3wwka; https://modelarchive.org/).

### Cells

HEK 293T (human embryo kidney cells; ATCC: CRL-11268), MDBK (Mardin Darby Bovine Kidney; ATCC: CRL-6071), and BEK (bovine embryo kidney; Istituto Zooprofilattico Sperimentale, Brescia, Italy; BS CL-94) were cultured in complete Eagle’s minimal essential medium (cEMEM). cEMEM was supplemented with 2 mM of L-glutamine, 1 mM of sodium pyruvate, 100 IU/mL of penicillin, 100 µg/mL of streptomycin, 0.25 µg/mL of amphotericin B, and 10% FBS. Cells were cultured in a humidified incubator at 37°C/5% CO_2_. All the supplements for the culture medium were purchased from Gibco (Segrate, MI, Italy).

### Construct generation

CpHV-1 gD secreted fragment (Sec-gD), lacking the transmembrane domain, was obtained by PCR amplification from pINT2-CMV-gDcpgD_106_ (20) with NheI-Cap-gD sense (5′-CCCGCTAGCATGTGGGCCCTCGTGCTCGCAGCGCTAAGC-3′) and BamHI-HA-Cap-gD antisense (5′-GGGGGATCCTTAGGCGTAATCGGGCACGTCGTAGGGGTACGGCGCGGCGGGCGGGAGGGTAGGC- 3′) primers. This pair of primers introduced an NheI restriction site at the 5′ terminal and an HA tag of the open reading frame (ORF), followed by a BamHI site at the 3′ terminal. The PCR amplification reaction was implemented in a final volume of 50 µL, containing 20 mM Tris–hydrochloride pH 8.8, 2 mM MgSO_4_, 10 mM KCl, 10 mM (NH_4_)_2_SO_4_, 0.1 mg/mL bovine serum albumin (BSA), 0.1% (vol/vol) Triton X-100, 5% dimethyl sulfoxide (DMSO), 0.2 mM deoxynucleotide triphosphate, and 0.25 µM of each primer. One unit of Pfu recombinant DNA polymerase (Thermo Fisher Scientific, Waltham, MA, USA) was used to amplify 100 ng of template DNA over 35 repeated cycles, including 1 min of denaturation at 94°C, 1 min of annealing at 60°C, and 1 min of elongation at 72°C. The resulting Sec-gD-HA amplicon was restriction digested with NheI/BamHI and subcloned in pEGFP-C1 (Clontech, San Jose, CA, USA), previously digested with the same enzymes, to generate the pCMV-Sec-gD construct.

### Transient transfection

HEK 293T cells were plated into 175 cm^2^ flasks (5 × 10^6^ cells/flask) and incubated at 37°C with 5% CO_2_. At sub-confluent density, the culture medium was removed, and the cells were transfected with pCMV-Sec-gD or pEGFP-C1 (as a mock control; Clontech, San Jose, CA, USA) using polyethylenimine (PEI) transfection reagent (Polysciences, Inc., Warrington, PA, USA). Briefly, DNA was mixed with PEI in a ratio of 1:2.5 (DNA:PEI) in 3.500 mL of serum-free Dulbecco’s modified essential medium (DMEM) with high glucose (Euroclone, Pero, Italy) and incubated for 15 min at room temperature. Next, 4× volumes of serum-free medium were added, and the transfection solution was transferred onto the cells monolayer and left for 6 h at 37°C with 5% CO_2_, in a humidified incubator. The transfection mixture was then replaced with 21 mL of DMEM/F12 (Ham’s F12 Nutrient Mixture; Euroclone Pero, Italy) (1:1) and incubated for 48 h. The cell supernatants, containing Sec-gD protein, were then harvested, clarified at 2,500 rpm at 4°C and stored at −80°C.

### Immunoblotting

Different amounts of Sec-gD protein supernatant samples were electrophoresed on 10% SDS-PAGE after total protein quantification with a BCA Protein Assay Kit (Pierce, Thermo Scientific, Waltham, MA, USA) and then transferred to PVDF membranes by electroblotting (Millipore, Merck, Rahway, NJ, USA). The membrane was subsequently blocked in 5% skim milk (Becton Dickinson, San Jose, CA, USA), incubated for 1 h at RT with a primary mouse monoclonal antibody anti-HA tag (G036, Abm Inc., New York, NY, USA), diluted at 1:15,000, and then probed with horseradish peroxidase-labeled anti-mouse immunoglobulin (A9044, Sigma-Aldrich [Merck], Tokyo, Japan), diluted at 1:15,000, and visualized using enhanced chemiluminescence (Clarity Max Western ECL substrate, Bio-Rad, Hercules, CA, USA).

### Peptide-N-glycosidase F digestion

Peptide-N-glycosidase F (PNGase F) (New England BioLabs, Ipswich, MA, USA) was employed following the manufacturer’s instructions. Sec-gD protein-containing supernatants were collected from pCMV-Sec-gD transiently transfected HEK cells after 48 h of secretion. The samples were then treated with PNGase F, which cleaves between the innermost N-acetylglucosamine (GlcNAc) and asparagine residues from N-linked glycoproteins. PNGase F-treated samples were subsequently analyzed by Western immunoblotting as described above.

### Site-directed mutagenesis

N-glycosylation-mutated Sec-gD variants were generated by site-directed mutagenesis using PCR with primers carrying the desired point mutations that removed N-glycosylation sites (File S5). Briefly, PCR was carried out in a final volume of 25 µL containing 20 mM Tris–HCl (pH 8.8), 2 mM MgSO_4_, 10 mM KCl, 10 mM (NH_4_)_2_SO_4_, 0.1 mg/mL BSA, 0.1% (vol/vol) Triton X-100, 10% (vol/vol) DMSO, 0.2 mM dNTPs, and 0.25 µM of each primer. One unit of recombinant Pfu DNA polymerase (Thermo Fisher Scientific, Waltham, MA, USA) was used to amplify 100 ng of template DNA over 35 cycles of 1 min denaturation at 94°C, 1 min annealing at 60°C, and 3 min extension at 72°C. PCR amplicons were separated by agarose gel electrophoresis and purified using the GeneJET Gel Extraction Kit (Thermo Fisher Scientific, Waltham, MA, USA) according to the manufacturer’s instructions. The purified DNA was mixed at a 1:2 (vol/vol) ratio with Gibson GeneArt HiFi Master Mix (Thermo Fisher Scientific, Waltham, MA, USA), incubated for 40 min at 50°C, then cooled and transformed into ElectroMAX DH10B electrocompetent *Escherichia coli* cells. Positive colonies were identified by plasmid DNA mini-preparation and restriction enzyme digestion, followed by transient transfection into HEK 293T cells and Western immunoblotting as described above.

### Virus titration

BA-1 strain of CpHV-1, isolated from a latently infected goat, was cultured and titrated in MDBK cells (4). The stock viral titer of 10^6.25^ 50% tissue culture infectious doses (TCID_50_)/50 µL was stored at −80°C and used for the experiments. Briefly, the stock virus was serially 10-fold diluted and inoculated in quadruplicate onto MDBK cells in 96-well microtiter plates and incubated at 37°C in a 5% CO_2_ atmosphere environment. The result was read after 3 days of incubation, and viral titers were expressed as logarithmic units calculated by the Reed-Muench method (21).

### Animals and experimental infection

The experimental protocol for goat infection was duly authorized (code 48E68, min aut. 869/15.11.2021) and conducted at the authorized University of Bari experimental animal facility (authorization no. 06/2023-UT).

Ten 5-year-old female goats without neutralizing antibodies to CpHV-1, as demonstrated by seroneutralization assay (SNA), were used in this study. Prior to experimentation, the goats were held under controlled environmental conditions and examined daily for clinical evidence of disease. Nasal and vaginal swabs from each goat were collected at the time the goats arrived at the laboratory, and just before inoculation to ensure the absence of ongoing infection.

Goats were intravaginally infected with the BA-1 strain of CpHV-1, each receiving 3.0 mL (2 × 10^6^ TCID_50_/mL) of the virus preparation and were daily examined for clinical evidence of infection. Observed clinical signs such as hyperemia, edema, lesions, and pain were scored as 0 (absent), 1 (mild), 2 (moderate), and 3 (severe), respectively. Temperature elevations above normal (38.2°C–38.6°C) were graded as 1 (>0.5°C to 1°C), 2 (1.1°C–1.5°C), and 3 (>1.5°C). The score for each animal was monitored and reported daily. Blood samples were taken at day 0 and at 42 days post-infection to evaluate antibody response to CpHV-1 by means of SNA. Vaginal swabs were also obtained daily for 11 days post-infection to evaluate virus shedding.

### Serum neutralization test

The serum neutralization (SN) assay, yielding the highest degree of specificity of all the serological tests, was used to assess seroconversion following the procedure described elsewhere (22). In brief, sera were obtained from goats by venipuncture in EDTA-free vacutainer and heat inactivated at 56°C for 30 min. Subsequently, serial twofold dilutions of each serum from 1:2 up to 1:32 were mixed with 100 TCID_50_ of the BA-1 strain of CpHV-1 in 96-well microtiter plates. The plates were held for 45 min at room temperature, and then, 20,000 MDBK cells in a volume of 0.05 mL of DMEM were added to each well, and the plates were then incubated for 3–5 days at 37°C in a 5% CO_2_ humidified chamber environment. Readings were made when CPEs were complete in the virus control cultures, and the titer of each serum was expressed as the highest dilution neutralizing the virus in the well.

### Samples collection and ELISA procedure

Ninety-six-well microplates (Microlon High Binding, Greiner Bio-One, Kremsmünster, Austria) were coated overnight at 4°C with 50 ng/well of Sec-gD protein supernatant diluted in 0.1 M carbonate/bicarbonate buffer at pH 9.6. After blocking with 1% BSA (Sigma Aldrich by Merck, Rome, Italy), goat serum samples at different twofold dilutions (1/10, 1/20, 1/40, 1/80, 1/160, 1/320, 1/640, and 1/1,280) were incubated for 1 h at room temperature. Serum samples were diluted in DMEM/F12 without serum, collected from HEK 293T grown for 48 h. After three washing steps in phosphate buffer saline, 50 µL of donkey anti-goat IgG-HRP (Santa Cruz Biotechnology, Heidelberg, Germany) diluted 1:5,000 was added to each well, and the plate was incubated as above. Following the final washing step, the reaction was developed with 3,3′,5,5′-tetramethylbenzidine (Merck, Rome, Italy), stopped with 0.2 M H_2_SO_4_ and read at 450 nm.

### Reverse serum neutralization test

Three heat-inactivated caprine sera previously confirmed to be positive in SNAs against CpHV-1 were selected. Twenty-five microliters of each CpHV-1 neutralizing serum sample were added to the first row of 96-well plates. An equal volume of cMEM (without FBS) was added to each well, and for each serum tested, serial twofold dilutions were performed. Next, 25 µL of medium containing Sec-gD protein was added to each well. Each serum was tested in the presence (+Sec-gD) or in the absence of Sec-gD (−Sec-gD). Positive and negative virus controls were also included. After 1 h of incubation at room temperature, 25 µL of virus suspension containing 100 TCID_50_ (50% tissue cell infectious doses) of CpHV-1 strain BA-1 (23) was added to each well. After 1 h of incubation at 37°C, 50 µL of a BEK cell suspension (2 × 10^5^ cells/mL) was added to each well, and the plates were incubated for 2 days at 37°C/5% CO_2_. Expression of viral infectivity and serum neutralizing activity through CPE was detected by microscopy and/or by classical crystal violet staining of the cell monolayer. The neutralization antibody titer was expressed as the reciprocal (log_2_) of the final dilution of serum that completely inhibited viral infectivity.

### Receiver operating characteristic analysis

Receiver operating characteristic (ROC) analysis was carried out using SPSS for Windows (version 29.0.1.0, SPSS Inc., Chicago, USA), and the results were plotted using GraphPad Prism (version 8.0.1, GraphPad Software Inc., Boston, USA).

## RESULTS

### Generation and expression of CpHV-1 gD as a secreted peptide

**Fig 1:**
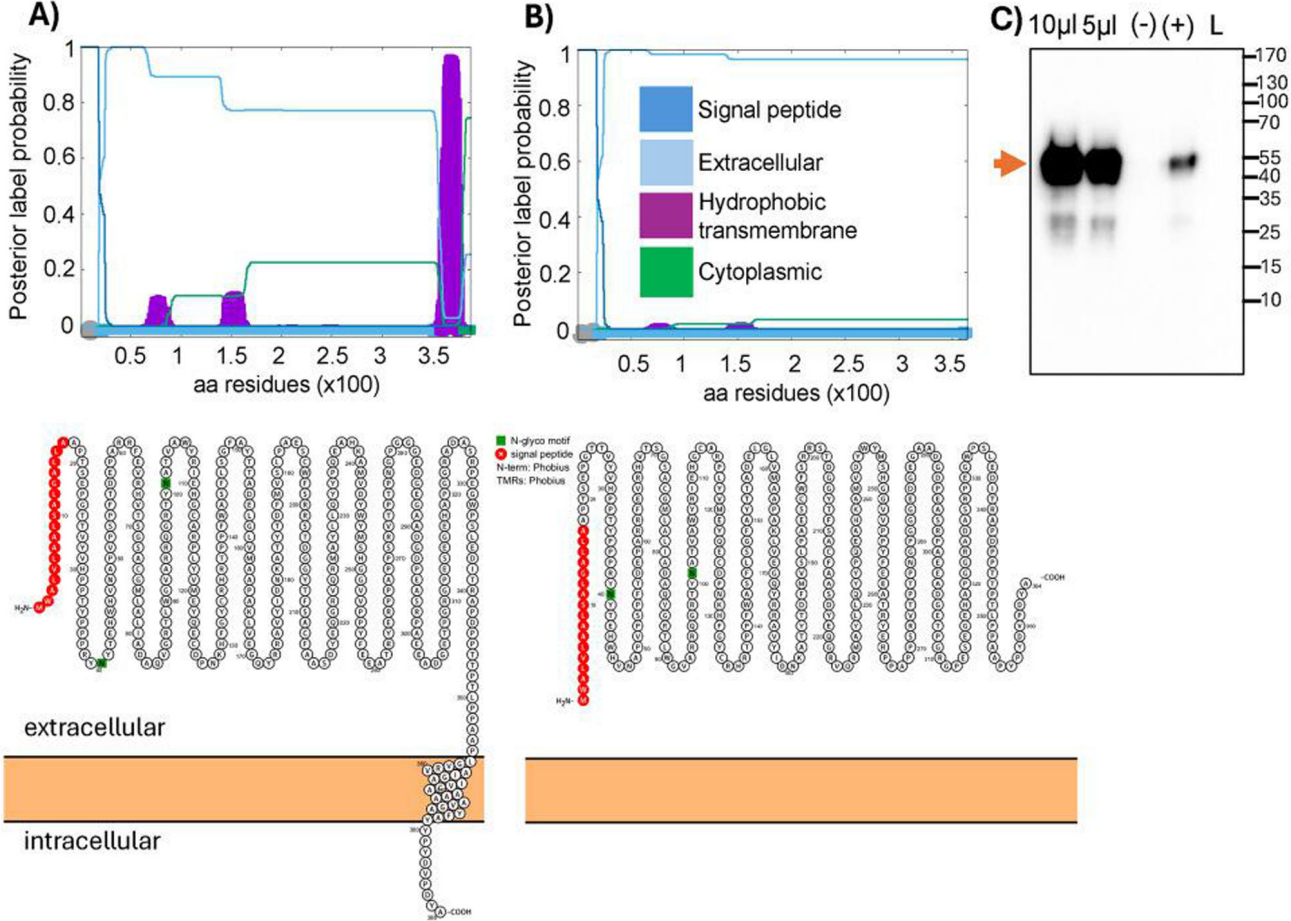
CpHV-1 Sec-gD analysis and expression. Phobius (top panel) and Protter (bottom panel) server output prediction of transmembrane topology and signal peptides from the amino acid sequence of gD (**A**) and Sec-gD proteins (**B**), respectively. The plot shows the posterior probabilities of cytoplasmic (green)/extracellular (azure)/TM helix (purple)/signal peptide (blue) by calculating the total probability that a residue belongs to a helix, cytoplasmic, or non-cytoplasmic summed over all possible paths through the model. (**C**) Western immunoblotting of Sec-gD coming from transfected HEK 293T cells supernatant. Lanes were loaded with 10 µL and 5 µL of serum-free medium transfected HEK 293T cells supernatant. Sec-gD showed a molecular mass of 55–60 kDa. Negative control was established with pEGFPC-1 serum-free medium transfected HEK 293T cells supernatant (−), whereas positive control was established with pCMV-E2-HA (an HA tagged unrelated protein), transfected HEK 293T cells serum-free medium supernatant (+), mass ladder (L).

**Fig 2:**
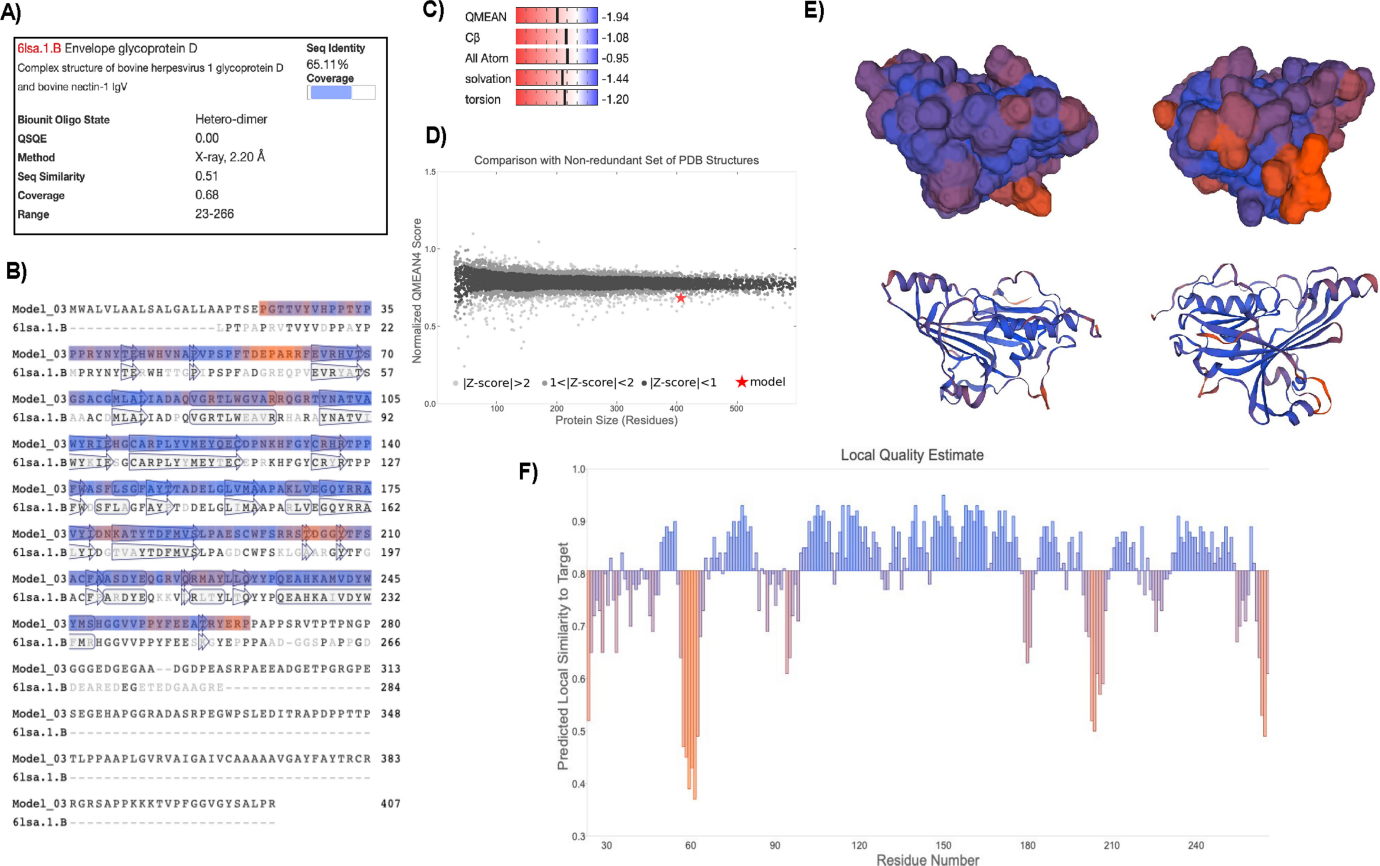
Homology modeling of CpHV-1 gD using SWISS-MODEL. The 3D structure of CpHV-1 gD extracellular domain was predicted (residues 23–266) using the crystal structure of BoHV-1 gD (PDB ID: 6lsa.1.B) as a template. (**A**) Template details including sequence identity (65.11%), coverage (68%), experimental determination method, and resolution. (**B**) Sequence alignment of the predicted CpHV-1 gD model with the BoHV-1 gD template, highlighting aligned residues and conserved secondary structures (arrows: β-strands and cylinders: α-helices). (**C**) Global quality estimates using QMEAN scoring. The overall QMEAN score (−1.94) indicates that the predicted model is of acceptable quality and comparable to experimentally determined protein structures of similar size. Individual QMEAN components are also shown: Cβ (−1.08), all-atom contacts (−0.95), solvation (−1.44), and torsion (−1.20). All scores are within a reasonable range, supporting the reliability of the model. (**D**) QMEAN *Z*-score plot comparing the model to a non-redundant set of PDB structure with similar size. (**E**) 3D structure of the CpHV-1 gD computed model on two different orientations. (**F**) QMEANDisCo Local quality estimates per residue, with predicted local similarity to the template (QMEANDisCo Global: 0.81 ± 0.05). Residues are colored based on predicted structural reliability (blue for well-conserved and confidently modeled regions, and orange for areas of lower structural confidence).

Based on its amino acid sequence and predictions from Phobius/Protter (http://phobius.sbc.su.se/), which are consistent with findings by Keuser et al. (12), the CpHV-1 gD ORF is 1,224 nucleotides long and encodes a 407-amino acid protein. This protein has a predicted molecular mass of 42.6 kDa and includes a signal peptide (amino acids 1–17), a hydrophobic transmembrane domain (amino acids 360–376) near the C-terminus, and a 31-amino acid cytoplasmic tail (Fig. 1A; File S1). The full-length gD can be expressed in eukaryotic systems as a membrane-bound protein (20). Based on this structure, removal of the transmembrane domain was predicted to yield a secreted form of gD (Fig. 1B; File S1). For achieving this, the extracellular domain of gD was amplified by PCR using primers, with the antisense primer incorporating an HA tag sequence in-frame with the amino-terminal rest of the protein (File S1). The resulting ORF was cloned into an expression vector, generating pCMV-Sec-gD. This construct includes the CMV promoter, the gD ORF lacking the transmembrane domain fused to an HA tag, and the bovine growth hormone polyadenylation signal. Upon transfection into HEK 293T cells, Sec-gD was efficiently secreted into the culture supernatant (Fig. 2C). Although the predicted molecular weight of Sec-gD is 40 kDa, it appears between 55 and 60 kDa in Western blot analysis, likely due to post-translational modifications such as glycosylation, as previously reported (12).

### Sec-gD structure analysis

While structural data for gD proteins from other alpha herpesviruses are available, no such information currently exists for CpHV-1. To address this gap, the secondary structure of CpHV-1 gD was predicted using two computational tools. GOR IV (accessed on 28 May 2025) estimated the protein to consist of 61.43% random coil, 20.15% alpha-helix, and 18.43% extended strand. Similarly, PSIPRED 4.0 (accessed on 28 May 2025) predicted 71.79% random coil, 19.27% alpha-helix, and 8.94% extended strand (File S2). To model the 3D structure of CpHV-1 gD, template-based modeling was performed using the SWISS-MODEL web server (accessed on 28 May 2025). Among 172 matching templates, 10 were selected based on sequence coverage (0.53–0.68), identity (43.32%–70.27%), and GMQE scores (0.39–0.51), all derived from previously resolved alpha herpesvirus gD structures (File S3). The final model was built using the bovine herpesvirus 1 gD structure (PDB ID: 6lsa.1.B) (24), which shares 65.11% sequence identity and 68% coverage with CpHV-1 gD. The resulting model spans residues 23 to 266, encompassing the majority of the extracellular domain (Fig. 2A). Model quality assessment yielded a GMQE of 0.51, a QMEANDisCo Global score of 0.81 ± 0.05, and a QMEAN *Z*-score of −1.94 (Fig. 2C), indicating that the predicted structure is reasonably accurate and falls within the range of experimentally determined proteins of similar size (Fig. 2D). Structural visualization and local quality estimation (Fig. 2E and F) showed high confidence in most surface-exposed regions, with lower reliability observed in loop regions around residues ~55–65 and ~200–210. To further refine the model, GalaxyRefine was employed. Both the initial and refined models were evaluated using the SWISS-MODEL structure assessment tool (accessed on 28 May 2025). Ramachandran plot analysis confirmed the structural validity, with the refined model showing improved accuracy: 98.35% of residues were in favored regions with 0.00% outliers, compared to 95.04% favored and 0.83% outliers in the crude model (File S4).

### Sec-gD exhibits conserved antigenic traits

**Fig 3:**
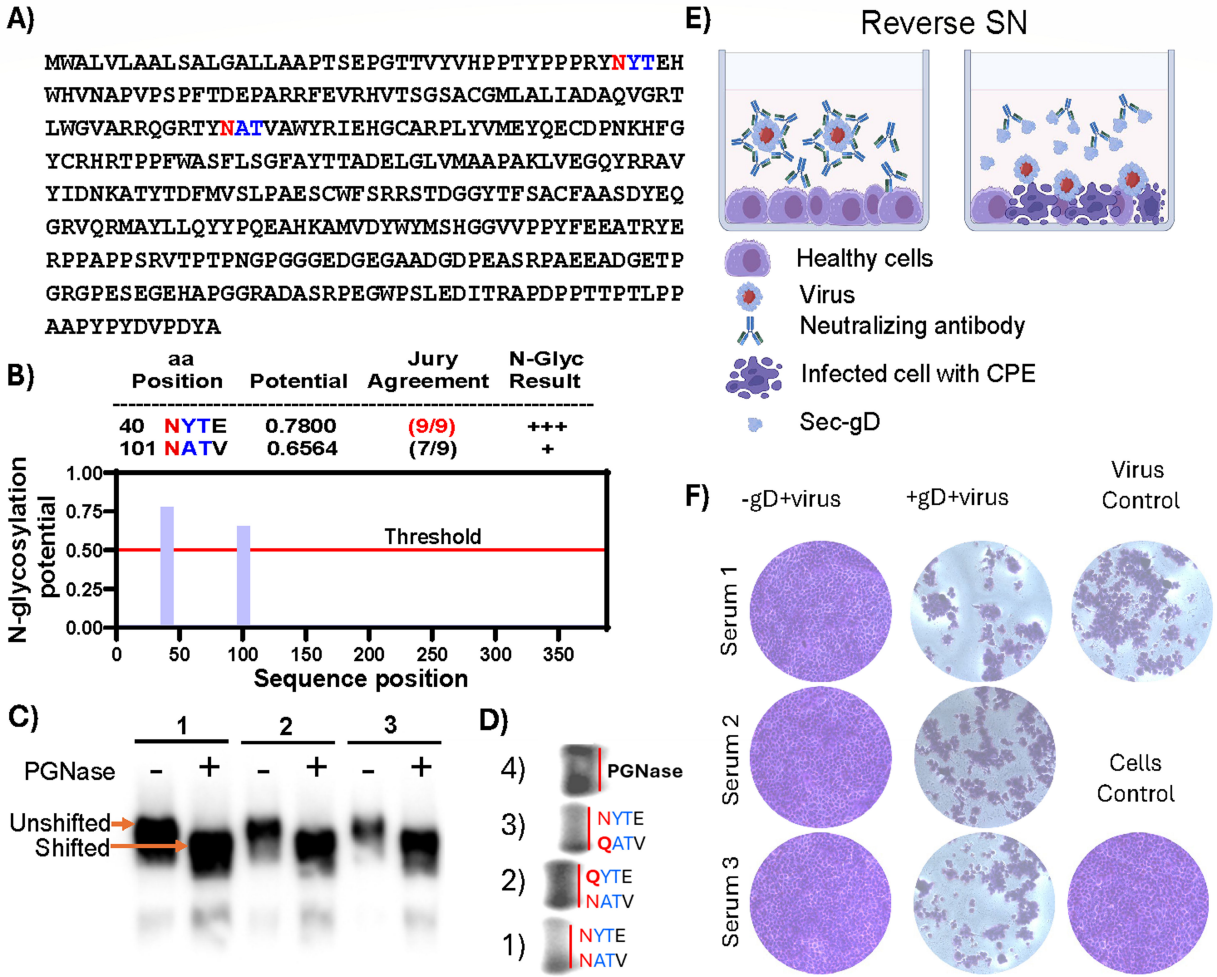
Sec-gD antigenic traits. (**A**) Sec-gD amino acids sequence where N-linked glycosylation sites are predicted by NetGlyc 1.0. (**B**) Potentially glycosylated asparagines (N) are in red and flanked by consensus amino acids in blue. The value crossing the default threshold of 0.5 represents a predicted glycosylated site (as long as it occurs in the required sequence Asn-Xaa-Ser/Thr without Proline at Xaa). The “potential” score is the averaged output of nine neural networks. For further information, the jury agreement column indicates how many of the nine networks support the prediction. (**C**) Western immunoblotting of Sec-gD protein treated (+) or untreated (−) with PGNase F. The assay was repeated three times (1, 2, and 3) with different amounts of protein to have a better resolution in terms of molecular size difference between glycosylated (unshifted) and deglycosylated (shifted) Sec-gD. (**D**) Western blot analysis of (1) unmutated Sec-gD (with predicted N-linked glycosylation sites NYTE and NATV), (2) Sec-gD mutated at the NYTE site (asparagine replaced by glutamine), (3) Sec-gD mutated at the NATV site (asparagine replaced by glutamine), and (4) fully deglycosylated Sec-gD treated with PNGase. Red lines indicate the migration front in the gel, highlighting differences in electrophoretic mobility among the samples. (**E**) Reverse SN assay to test the authentic antigenic characteristics of Sec-gD. Cartoon explaining the rationale of the assay, where CpHV-1 neutralizing sera preincubated with medium containing Sec-gD are blocked by Sec-gD interaction and allowing the virus to induce CPE on MDBK cells. Created in BioRender (biorender.com). (**F**) The quantitative assay performed in a 96-multiwell plate, three sera (Serum 1, 2 and 3) containing neutralizing antibodies against CpHV-1 were tested at the dilutions of 1/10 in the presence of Sec-gD (+Sec-gD) and in the absence of Sec-gD (−Sec-gD). Virus control was established in the absence of sera and Sec-gD (virus control) and a cells control with cells without virus, sera, and Sec-gD (cell control). Crystal violet staining allows macroscopic (not shown) and microscopic evaluation of the integrity (violet wells) of the cell monolayer.

The gD glycoprotein plays a crucial role in the attachment and entry of CpHV-1 into host cells (12). Additionally, CpHV-1 gD is essential for eliciting neutralizing antibodies and conferring protection against experimental CpHV-1 infection in goats (20, 22). Protein glycosylation can significantly influence antibody function, particularly affecting antigen recognition and binding affinity. This is especially relevant for conformational antibodies, which depend on the three-dimensional structure of the antigen and are therefore sensitive to glycosylation-induced changes (25). The gD is a glycosylated protein (12), including its secreted form (Sec-gD), which contains two predicted N-glycosylation sites at amino acid positions 40 and 101 (Fig. 3A and B). These sites were experimentally confirmed through PNGase treatment and Western blot analysis (Fig. 3C). Replacing asparagine (N; red) with glutamine (Q; red) at the predicted glycosylation sites (**N**YTE and **N**ATV) (Fig. S5) caused a progressive shift in the protein’s molecular size, as observable on the protein band front electrophoretic mobility, relative to the unmutated form (fully glycosylated larger size) and the PNGase-digested form (fully deglycosylated smaller size) (Fig. 3D), thereby supporting the accuracy of the prediction.

Since structural prediction and analysis defined a certain degree of authenticity respect to other alpha herpesvirus glycoprotein D, it was of interest to ensure that Sec-gD retained its antigenic properties despite modifications such as removal of the transmembrane domain and addition of a 9-amino acid tag. Therefore, a reverse serum neutralization assay was conducted (Fig. 3E). As shown in Fig. 3F, preincubation of neutralizing sera with Sec-gD abolished their neutralizing activity, allowing CpHV-1 to infect and destroy the cell monolayer.

### Sec-gD allows the development of an indirect ELISA for the detection of CpHV-1-infected goats

**Fig 4:**
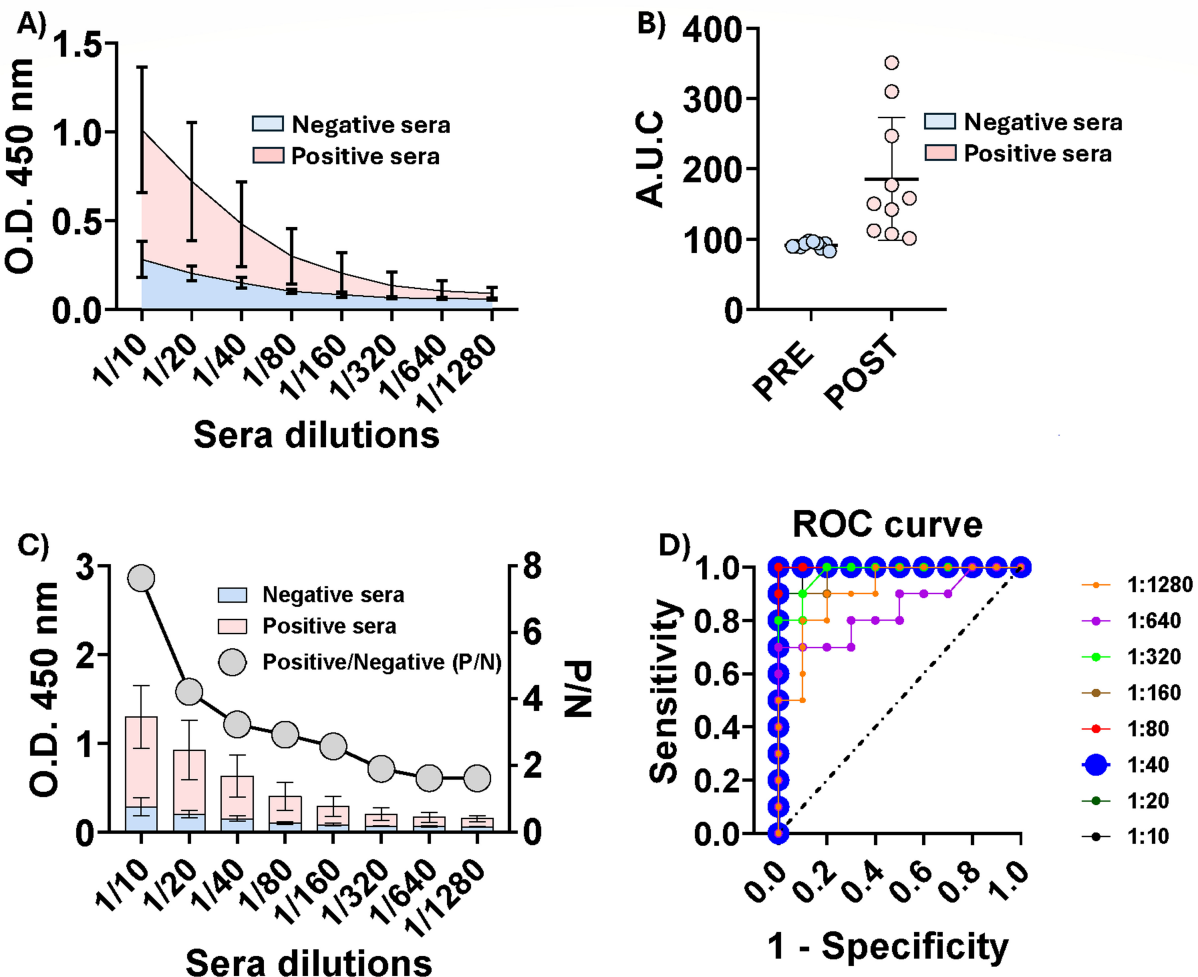
Reactivity of positive and negative sera toward Sec-gD. (**A**) Average reactivity of positive (pink) and negative (azure) sera at different dilutions. Differences were statistically significant for each dilution. (**B**) Data from the same experiment as in (**A**) but plotted as AUC to obtain a better quantitative impression. Statistical analyses were performed using an unpaired two-tailed Student’s *t*-test in GraphPad Prism (*P* < 0.0001). (**C**) Optimization of sera dilution, defined as the dilution yielding the highest ratio between the average signal of positive and negative sera (P/N). Differences were statistically significant for each dilution. (**D**) ROC analysis to determine the cut-off value of the test.

Sec-gD was used to coat 96-well ELISA plates, and ELISA assays were conducted using serial dilutions of individual serum samples. These included 10 sera from animals that tested negative by SN prior to experimental infection, and 10 sera collected from the same animals 42 days post-CpHV-1 infection, which tested SN-positive, were clinically symptomatic, and virologically shedding as monitored by clinical score from day 0 to day 11 post-infection (data not shown). The resulting dilution curves (Fig. 4A) and the corresponding average areas under the curves (Fig. 4B) allowed for clear discrimination between positive and negative sera. The serum dilution yielding the highest ratio between the average signal of positive and negative sera (P/N) was determined to be 1/40 (Fig. 4C). ROC analysis (Fig. 4D) confirmed that 1/40 could represent the optimal serum dilution. Indeed, as shown by the area under the curve (AUC) values reported in Table 1, both the 1/40 and 1/80 dilutions gave an AUC value of 1. However, the maximum Youden index was obtained with different cut-off points: 0.199 for 1/40 and 0.127 for 1/80 (File S6). Therefore, the dilution with the higher cut-off was chosen, since for a diagnostic purpose, a higher threshold is generally more robust against inaccurate readings at low absorbance levels. This helps to prevent false positives resulting from background noise or potential weak cross-reactivity.

**TABLE 1:** AUC values obtained at different dilutions of the tested sera^*a*^

Serum dilution	AUC	Standard error	*P* value	95% confidence interval	
				Lower limit	Upper limit
1/10	0.990	0.016	<0.001	0.958	1.022
1/20	0.980	0.026	<0.001	0.929	1.031
**1/40**	**1.000**	**0.000**	**<0.001**	**1.000**	**1.000**
**1/80**	**1.000**	**0.000**	**<0.001**	**1.000**	**1.000**
1/160	0.970	0.032	<0.001	0.907	1.033
1/320	0.975	0.029	<0.001	0.919	1.031
1/640	0.845	0.092	<0.001	0.665	1.025
1/1,280	0.910	0.065	<0.001	0.783	1.037

^*a*^ Sera dilutions with the best AUC are highlighted in bold.

## DISCUSSION

The gD of several alpha herpesviruses plays a key role in the viral entry process, which typically occurs in two stages: initial attachment, where viral attachment proteins interact with receptors on the host cell surface, followed by membrane fusion that enables viral penetration (26). Antibodies generated against gD can neutralize the virus, and the presence of these neutralizing antibodies in vaccinated animals and humans is considered a more reliable indicator of vaccine efficacy compared to cell-mediated immunity (27, 28). Despite truncated gD being used in human herpesviruses vaccine candidates with mixed results (29), no studies have yet explored the potential use of CpHV-1 gD or its secreted form as a viable antigen for diagnostics and vaccine development. In this study, Sec-gD was successfully obtained and characterized. The *in silico*-derived structure of Sec-gD displays features consistent with those of gD proteins from other alpha herpesviruses, including its glycosylation pattern. Biochemical analyses confirmed that Sec-gD is properly glycosylated. SDS-PAGE and Western blot analyses revealed that Sec-gD exhibits a higher molecular weight than predicted based on its amino acid sequence, a discrepancy that was resolved upon treatment with PNGase F and site-directed mutagenesis of the glycosylation sites, indicating the presence of N-linked glycans. Glycans often dominate the surface of viral glycoproteins, making the viral glycome a key factor in shaping the antigenicity and immunogenicity. At one end of the spectrum, glycans can form essential components of epitopes recognized by neutralizing antibodies, thus playing a critical role in the design of effective immunogens. Conversely, the success of peptide-based and bacterially expressed protein vaccines demonstrates that viral glycosylation is not always essential. Nevertheless, native-like glycosylation can reflect proper protein folding and the presence of conformational epitopes. Moreover, strategic modifications beyond native glycan mimicry—such as altering glycosylation site occupancy or glycan processing—may enhance the immunogenicity and protective efficacy of vaccine antigens (30). Given that proper protein folding and glycosylation can indicate the presence of conformational epitopes involved in the induction and recognition of neutralizing antibodies, this was validated through a reverse neutralization assay (30). In this test, Sec-gD effectively inhibited the CpHV-1 neutralizing activity of sera from CpHV-1-infected animals, confirming its role in antibody recognition. For corroborating the antigenic properties of CpHV-1 Sec-gD expressed in mammalian cells, an indirect ELISA test was developed. The ELISA test demonstrated complete accuracy in distinguishing sera from infected and non-infected animals.

This study utilized a secreted form of the gD protein (Sec-gD), expressed in mammalian cells. The Sec-gD protein demonstrated excellent immunogenicity, highlighting its potential not only for diagnostic applications but also as a subunit vaccine capable of inducing strong neutralizing antibody responses. Its performance underscores the importance of developing effective immunogens for successful vaccine strategies. Previous research identified the CpHV-1 gD protein as a promising vaccine candidate due to its critical role in viral attachment and entry into host cells. Recent findings confirm that this glycoprotein elicits potent neutralizing antibody responses, reinforcing its value as a key target for vaccine development. Animal studies further support this, showing that gD-based vaccines can confer protective immunity. A recombinant BoHV-4-based vector vaccine expressing the full-length CpHV-1 gD has recently been developed and shown to provide robust protection against lethal CpHV-1 infection in goats. The Sec-gD platform represents an evolution of this approach, offering greater flexibility. It can be adapted into various formats, such as a ferritin-based self-assembling nanoparticle displaying the Sec-gD head domain (Sec-gD-ferritin) or as nucleic acid vaccines (DNA or mRNA). These formats offer several advantages, including rapid development, low-cost manufacturing, scalability, stability at lower temperatures, and suitability for deployment in outbreak-prone regions. Notably, DNA or mRNA-based Sec-gD vaccines are expected to elicit high levels of IFN-γ from T cells and induce strong gD-specific IgG antibody responses. Moreover, Sec-gD can be engineered into different structural configurations—monomeric, multimeric, or chimeric subunits—and stabilized in either fusion or post-fusion forms, enhancing its versatility as a vaccine component.

In conclusion, the promising results and proof-of-concept for using Sec-gD presented in this study lay the groundwork for developing and testing additional derivatives as prototype diagnostic tools and vaccines. These could target other significant animal pathogens—and potentially even human pathogens—given that CpHV-1 serves as a valuable large animal model for studying varicella-zoster virus in humans.

## Data Availability

All data are available in this paper and Supplementary Materials, which can be retrieved by clicking the dedicated link.

## SUPPLEMENTAL MATERIAL

File S1 spectrum.02373-25-s0001.docx Caprine herpesvirus 1 annotated glycoprotein.

File S2 spectrum.02373-25-s0002.docx Secondary structures prediction of CpHV-1 gD.

File S3 spectrum.02373-25-s0003.docx Top 10 of the 172 templates matching the CpHV-1 gD sequence on SWISS-MODEL.

File S4 spectrum.02373-25-s0004.docx Structural assessment of CpHV-1 gD models.

File S5 spectrum.02373-25-s0005.docx Sec-gD mutants' generation.

File S6 spectrum.02373-25-s0006.docx ROC curve coordinates.

ASM does not own the copyrights to Supplemental Material that may be linked to, or accessed through, an article. The authors have granted ASM a non-exclusive, world-wide license to publish the Supplemental Material files. Please contact the corresponding author directly for reuse.

## References

[1] Davison AJ. 2010. Herpesvirus systematics. Vet Microbiol 143:52–69. doi:10.1016/j.vetmic.2010.02.014 20346601 PMC2995426

[2] Van der Lugt JJ, Randles JL. 1993. Systemic herpesvirus infection in neonatal goats. J S Afr Vet Assoc 64:169–171. 8176698

[3] Tarigan S, Webb RF, Kirkland D. 1987. Caprine herpesvirus from balanoposthitis. Aust Vet J 64:321. doi:10.1111/j.1751-0813.1987.tb07345.x 2830872

[4] Tempesta M., Pratelli A, Corrente M, Buonavoglia C. 1999a. A preliminary study on the pathogenicity of a strain of caprine herpesvirus-1. Comp Immunol Microbiol Infect Dis 22:137–143. doi:10.1016/S0147-9571(98)00029-0 10051183

[5] Keuser V., Gogev S, Schynts F, Thiry E. 2002. Demonstration of generalized infection with caprine herpesvirus 1 diagnosed in an aborted caprine fetus by PCR. Vet Res Commun 26:221–226. doi:10.1023/a:1015205705149 12090293

[6] Engels M, Loepfe E, Wild P, Schraner E, Wyler R. 1987. The genome of caprine herpesvirus 1: genome structure and relatedness to bovine herpesvirus 1. J Gen Virol 68 (Pt 7):2019–2023. doi:10.1099/0022-1317-68-7-2019 3037021

[7] Hao F, Mao L, Li W, Li J, Yang L, Zhang W, Jiang J, Sun M, Xie X, Liu M. 2020. Epidemiological investigation and genomic characterization of Caprine herpesvirus 1 from goats in China. Infect Genet Evol 79:104168. doi:10.1016/j.meegid.2019.104168 31899234

[8] Uzal FA, Woods L, Stillian M, Nordhausen R, Read DH, Van Kampen H, Odani J, Hietala S, Hurley EJ, Vickers ML, Gard SM. 2004. Abortion and ulcerative posthitis associated with caprine herpesvirus-1 infection in goats in California. J Vet Diagn Invest 16:478–484. doi:10.1177/104063870401600523 15460339

[9] Tempesta M., Pratelli A, Greco G, Martella V, Buonavoglia C. 1999b. Detection of caprine herpesvirus 1 in sacral ganglia of latently infected goats by PCR. J Clin Microbiol 37:1598–1599. doi:10.1128/JCM.37.5.1598-1599.1999 10203533 PMC84844

[10] Camero M, Crescenzo G, Marinaro M, Tarsitano E, Bellacicco AL, Armenise C, Buonavoglia C, Tempesta M. 2010. Cidofovir does not prevent caprine herpesvirus type-1 neural latency in goats. Antivir Ther 15:785–788. doi:10.3851/IMP1611 20710060

[11] Schwyzer M, Ackermann M. 1996. Molecular virology of ruminant herpesviruses. Vet Microbiol 53:17–29. doi:10.1016/s0378-1135(96)01231-x 9010995

[12] Keuser Véronique, Detry B, Thiry J, de Fays K, Schynts F, Pastoret P-P, Vanderplasschen A, Thiry E. 2006. Characterization of caprine herpesvirus 1 glycoprotein D gene and its translation product. Virus Res 115:112–121. doi:10.1016/j.virusres.2005.07.009 16140410

[13] McGuffin LJ, Bryson K, Jones DT. 2000. The PSIPRED protein structure prediction server. Bioinformatics 16:404–405. doi:10.1093/bioinformatics/16.4.404 10869041

[14] Garnier J, Gibrat JF, Robson B. 1996. GOR method for predicting protein secondary structure from amino acid sequence. Methods Enzymol 266:540–553. doi:10.1016/s0076-6879(96)66034-0 8743705

[15] Käll L, Krogh A, Sonnhammer ELL. 2007. Advantages of combined transmembrane topology and signal peptide prediction--the Phobius web server. Nucleic Acids Res 35:W429–32. doi:10.1093/nar/gkm256 17483518 PMC1933244

[16] Omasits U, Ahrens CH, Müller S, Wollscheid B. 2014. Protter: interactive protein feature visualization and integration with experimental proteomic data. Bioinformatics 30:884–886. doi:10.1093/bioinformatics/btt607 24162465

[17] Gupta R, Brunak S. 2002. Prediction of glycosylation across the human proteome and the correlation to protein function, p 310–322. Pac Symp Biocomput. 11928486

[18] Waterhouse A, Bertoni M, Bienert S, Studer G, Tauriello G, Gumienny R, Heer FT, de Beer TAP, Rempfer C, Bordoli L, Lepore R, Schwede T. 2018. SWISS-MODEL: homology modelling of protein structures and complexes. Nucleic Acids Res 46:W296–W303. doi:10.1093/nar/gky427 29788355 PMC6030848

[19] Ko J, Park H, Heo L, Seok C. 2012. GalaxyWEB server for protein structure prediction and refinement. Nucleic Acids Res 40:W294–7. doi:10.1093/nar/gks493 22649060 PMC3394311

[20] Donofrio G, Franceschi V, Lovero A, Capocefalo A, Camero M, Losurdo M, Cavirani S, Marinaro M, Grandolfo E, Buonavoglia C, Tempesta M. 2013. Clinical protection of goats against CpHV-1 induced genital disease with a BoHV-4-based vector expressing CpHV-1 gD. PLoS One 8:e52758. doi:10.1371/journal.pone.0052758 23300989 PMC3536792

[21] Tempesta Maria, Camero M, Bellacicco AL, Thiry J, Crescenzo G, Neyts J, Thiry E, Buonavoglia C. 2007. Cidofovir is effective against caprine herpesvirus 1 infection in goats. Antiviral Res 74:138–141. doi:10.1016/j.antiviral.2006.11.001 17161474

[22] Tempesta M, Pratelli A, Normanno G, Camero M, Buonavoglia D, Greco G, Buonavoglia C. 2000. Experimental intravaginal infection of goats with caprine herpesvirus 1. J Vet Med B Infect Dis Vet Public Health 47:197–201. doi:10.1046/j.1439-0450.2000.00334.x 10829574

[23] Buonavoglia C, Tempesta M, Cavalli A, Voigt V, Buonavoglia D, Conserva A, Corrente M. 1996. Reactivation of caprine herpesvirus 1 in latently infected goats. Comp Immunol Microbiol Infect Dis 19:275–281. doi:10.1016/0147-9571(96)00014-8 8894377

[24] Yue D, Chen Z, Yang F, Ye F, Lin S, He B, Cheng Y, Wang J, Chen Z, Lin X, Yang J, Chen H, Zhang Z, You Y, Sun H, Wen A, Wang L, Zheng Y, Cao Y, Li Y, Lu G. 2020. Crystal structure of bovine herpesvirus 1 glycoprotein D bound to nectin-1 reveals the basis for its low-affinity binding to the receptor. Sci Adv 6:eaba5147. doi:10.1126/sciadv.aba5147 32426511 PMC7220272

[25] Tremain AC, Wallace RP, Lorentz KM, Thornley TB, Antane JT, Raczy MR, Reda JW, Alpar AT, Slezak AJ, Watkins EA, Maulloo CD, Budina E, Solanki A, Nguyen M, Bischoff DJ, Harrington JL, Mishra R, Conley GP, Marlin R, Dereuddre-Bosquet N, Gallouët A-S, LeGrand R, Wilson DS, Kontos S, Hubbell JA. 2023. Synthetically glycosylated antigens for the antigen-specific suppression of established immune responses. Nat Biomed Eng 7:1142–1155. doi:10.1038/s41551-023-01086-2 37679570

[26] Zhong L, Zhang W, Krummenacher C, Chen Y, Zheng Q, Zhao Q, Zeng M-S, Xia N, Zeng Y-X, Xu M, Zhang X. 2023. Targeting herpesvirus entry complex and fusogen glycoproteins with prophylactic and therapeutic agents. Trends Microbiol 31:788–804. doi:10.1016/j.tim.2023.03.001 36967248

[27] Alves Dummer L, Pereira Leivas Leite F, van Drunen Littel-van den Hurk S. 2014. Bovine herpesvirus glycoprotein D: a review of its structural characteristics and applications in vaccinology. Vet Res 45:111. doi:10.1186/s13567-014-0111-x 25359626 PMC4252008

[28] Georgopoulos AP, James LM. 2024. Immunogenetic profiles of 9 human herpes virus envelope glycoproteins. Sci Rep 14:20924. doi:10.1038/s41598-024-71558-1 39251790 PMC11385983

[29] Stanberry LR, Spruance SL, Cunningham AL, Bernstein DI, Mindel A, Sacks S, Tyring S, Aoki FY, Slaoui M, Denis M, Vandepapeliere P, Dubin G, GlaxoSmithKline Herpes Vaccine Efficacy Study Group. 2002. Glycoprotein-D-adjuvant vaccine to prevent genital herpes. N Engl J Med 347:1652–1661. doi:10.1056/NEJMoa011915 12444179

[30] Newby ML, Allen JD, Crispin M. 2024. Influence of glycosylation on the immunogenicity and antigenicity of viral immunogens. Biotechnol Adv 70:108283. doi:10.1016/j.biotechadv.2023.108283 37972669 PMC10867814

