# Supplementary material for: Generation, characterization, and application of caprine herpesvirus 1 secreted glycoprotein D
Source: Microbiol Spectr. 2025 Nov 28;14(1):e02373-25. doi: 10.1128/spectrum.02373-25 (PMC12772238; doi:10.1128/spectrum.02373-25)
Supplement: File S1 — Caprine herpesvirus 1 annotated glycoprotein. [file spectrum.02373-25-s0001.docx]

**Caprine Herpesvirus 1 glycoprotein D**

atgtgggccctcgtgctcgcagcgctaagcgcgctcggggcgctgctggccgcgccgacg

M W A L V L A A L S A L G A L L A A P T 20

tccgagcccgggacgaccgtgtatgtgcatccgccgacatacccgccgccgcggtacaac

S E P G T T V Y V H P P T Y P P P R Y N 40

tacacggagcactggcacgtcaacgccccggtcccgtcgccgtttaccgacgagcccgcg

Y T E H W H V N A P V P S P F T D E P A 60

cggcgctttgaggtgcgccacgtaacgagcggctcggcgtgcggcatgctggccctcatc

R R F E V R H V T S G S A C G M L A L I 80

gcggatgcccaggtggggcggaccctgtggggggtggcgcgccggcaggggcgcacgtac

A D A Q V G R T L W G V A R R Q G R T Y 100

aacgccacggtcgcgtggtaccggatagagcacggctgcgcccggccgctgtacgtgatg

N A T V A W Y R I E H G C A R P L Y V M 120

gagtaccaagagtgcgaccccaataagcactttggctactgccggcaccgcacccctccg

E Y Q E C D P N K H F G Y C R H R T P P 140

ttttgggccagctttctgtctgggttcgcgtacaccacggcggatgagctggggctcgtc

F W A S F L S G F A Y T T A D E L G L V 160

atggccgcgcccgcgaagctcgtcgagggccagtaccggcgggccgtgtacatcgacaac

M A A P A K L V E G Q Y R R A V Y I D N 180

aaggccacctacaccgacttcatggtctcgctgcccgcagagagctgctggttctctagg

K A T Y T D F M V S L P A E S C W F S R 200

cgcagcactgacggcgggtacaccttcagcgcctgcttcgcggcctcggactacgaacag

R S T D G G Y T F S A C F A A S D Y E Q 220

gggcgcgtgcagcggatggcgtacctcctccagtactacccccaagaggcgcacaaggcc

G R V Q R M A Y L L Q Y Y P Q E A H K A 240

atggtggattactggtacatgagccacgggggcgttgtgcccccgtacttcgaggaggcg

M V D Y W Y M S H G G V V P P Y F E E A 260

acgcgctacgagcgcccgccagcgccccccggccgcgtcacccccacgcccaatggcccg

T R Y E R P P A P P G R V T P T P N G P 280

gggggcggcgaagaaggcgagggggccgccgacggagacccggaggcgagccgccccgcg

G G G E E G E G A A D G D P E A S R P A 300

gaagaggcggacggcgagacccccggtcgcgggccagagagcgaaggcgaacacgccccg

E E A D G E T P G R G P E S E G E H A P 320

ggcggccgcgccgacgcgagccggcccgaaggctggccgagcctcgaagacatcacgcgg

G G R A D A S R P E G W P S L E D I T R 340

gcgccggacccgcctacgacgcctaccctcccgcccgccgcgccgctgggggtccgcgtc

A P D P P T T P T L P P A A P L G V R V 360

gcgattggcgccatcgtatgcgcggcggccgccgccgtcggcgcgtactttgcctacacg

A I G A I V C A A A A A V G A Y F A Y T 380

cgctgccggcgcgggcgcttggcgcccccaaagaaaaagacagtgccgttcggcggcgtc

R C R R G R L A P P K K K T V P F G G V 400

ggctacagcgcgctgccccgatga

G Y S A L P R - 407

SIGNAL 1 17

TOPO_DOM 18 355 NON CYTOPLASMIC.

TRANSMEM 356 379

TOPO_DOM 380 407 CYTOPLASMIC.

**Caprine Herpesvirus 1 Secreted glycoprotein D (Sec-gD)**

atg tgg gcc ctc gtg ctc gca gcg cta agc gcg ctc ggg gcg ctg ctg gcc gcg ccg acg 
 M   W   A   L   V   L   A   A   L   S   A   L   G   A   L   L   A   A   P   T  
tcc gag ccc ggg acg acc gtg tat gtg cat ccg ccg aca tac ccg ccg ccg cgg tac aac 
 S   E   P   G   T   T   V   Y   V   H   P   P   T   Y   P   P   P   R   Y   N  
tac acg gag cac tgg cac gtc aac gcc ccg gtc ccg tcg ccg ttt acc gac gag ccc gcg 
 Y   T   E   H   W   H   V   N   A   P   V   P   S   P   F   T   D   E   P   A  
cgg cgc ttt gag gtg cgc cac gta acg agc ggc tcg gcg tgc ggc atg ctg gcc ctc atc 
 R   R   F   E   V   R   H   V   T   S   G   S   A   C   G   M   L   A   L   I  
gcg gat gcc cag gtg ggg cgg acc ctg tgg ggg gtg gcg cgc cgg cag ggg cgc acg tac 
 A   D   A   Q   V   G   R   T   L   W   G   V   A   R   R   Q   G   R   T   Y  
aac gcc acg gtc gcg tgg tac cgg ata gag cac ggc tgc gcc cgg ccg ctg tac gtg atg 
 N   A   T   V   A   W   Y   R   I   E   H   G   C   A   R   P   L   Y   V   M  
gag tac caa gag tgc gac ccc aat aag cac ttt ggc tac tgc cgg cac cgc acc cct ccg 
 E   Y   Q   E   C   D   P   N   K   H   F   G   Y   C   R   H   R   T   P   P  
ttt tgg gcc agc ttt ctg tct ggg ttc gcg tac acc acg gcg gat gag ctg ggg ctc gtc 
 F   W   A   S   F   L   S   G   F   A   Y   T   T   A   D   E   L   G   L   V  
atg gcc gcg ccc gcg aag ctc gtc gag ggc cag tac cgg cgg gcc gtg tac atc gac aac 
 M   A   A   P   A   K   L   V   E   G   Q   Y   R   R   A   V   Y   I   D   N  
aag gcc acc tac acc gac ttc atg gtc tcg ctg ccc gca gag agc tgc tgg ttc tct agg 
 K   A   T   Y   T   D   F   M   V   S   L   P   A   E   S   C   W   F   S   R  
cgc agc act gac ggc ggg tac acc ttc agc gcc tgc ttc gcg gcc tcg gac tac gaa cag 
 R   S   T   D   G   G   Y   T   F   S   A   C   F   A   A   S   D   Y   E   Q  
ggg cgc gtg cag cgg atg gcg tac ctc ctc cag tac tac ccc caa gag gcg cac aag gcc 
 G   R   V   Q   R   M   A   Y   L   L   Q   Y   Y   P   Q   E   A   H   K   A  
atg gtg gat tac tgg tac atg agc cac ggg ggc gtt gtg ccc ccg tac ttc gag gag gcg 
 M   V   D   Y   W   Y   M   S   H   G   G   V   V   P   P   Y   F   E   E   A  
acg cgc tac gag cgc ccg cca gcg ccc ccc agc cgc gtc acc ccc acg ccc aat ggc ccg 
 T   R   Y   E   R   P   P   A   P   P   S   R   V   T   P   T   P   N   G   P  
ggg ggc ggc gaa gac ggc gag ggg gcc gcc gac gga gac ccg gag gca agc cgc ccc gcg 
 G   G   G   E   D   G   E   G   A   A   D   G   D   P   E   A   S   R   P   A  
gaa gag gcg gac ggc gag acc ccc ggt cgc ggg cca gag agc gaa ggc gaa cac gcc ccg 
 E   E   A   D   G   E   T   P   G   R   G   P   E   S   E   G   E   H   A   P  
ggc ggc cgc gcc gac gcg agc cgg ccc gaa ggc tgg ccg agc ctc gaa gac atc acg cgg 
 G   G   R   A   D   A   S   R   P   E   G   W   P   S   L   E   D   I   T   R  
gcg ccg gac ccg cct acg acg cct acc ctc ccg ccc gcc gcg ccg tac ccc tac gac gtg 
 A   P   D   P   P   T   T   P   T   L   P   P   A   A   P   Y   P   Y   D   V  
ccc gat tac gcc taa 
 P   D   Y   A   -

SIGNAL 1 17

TOPO_DOM 18 355 NON CYTOPLASMIC.

HA tag 356 364

**Supplementary File 2. Caprine herpesvirus 1 annotated glycoprotein.** Annotated nucleotides and amino acids sequences of CpHV-1 gD and Sec-gD.
