# Supplementary material for: Generation, characterization, and application of caprine herpesvirus 1 secreted glycoprotein D
Source: Microbiol Spectr. 2025 Nov 28;14(1):e02373-25. doi: 10.1128/spectrum.02373-25 (PMC12772238; doi:10.1128/spectrum.02373-25)
Supplement: File S2 — Secondary structures prediction of CpHV-1 gD. [file spectrum.02373-25-s0002.docx]

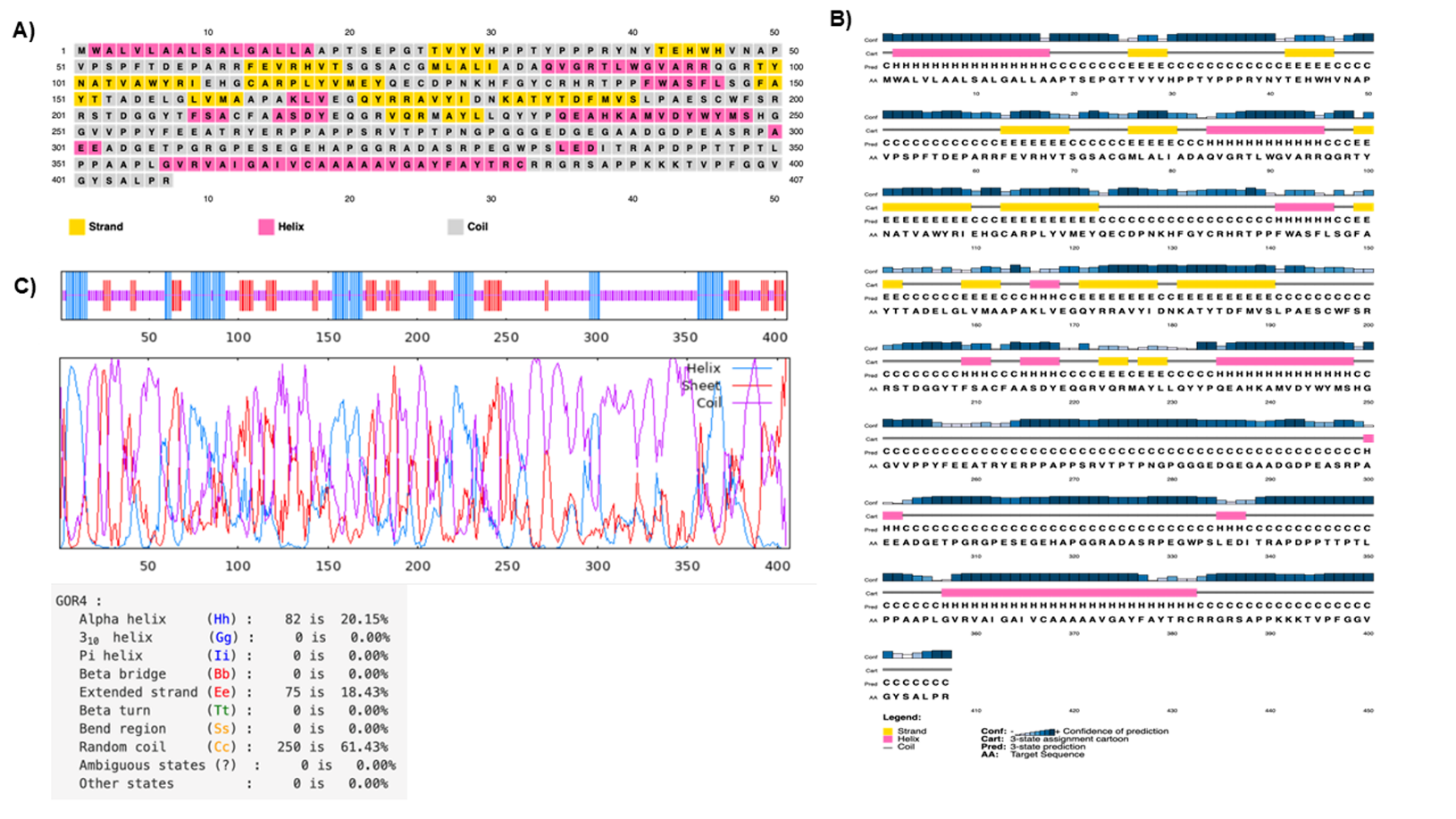


**Supplementary File 2. Secondary structures prediction of CpHV-1 gD**: **(A)** PSIPRED graphical output of secondary structure elements prediction (yellow: β-strands, pink: α-helices, grey: coils) and **(B)** predicted secondary structure along the sequence with associated prediction confidence scores. **(C)** GOR IV graphical output indicating predicted secondary structure elements along with probability values for each secondary structure —Helix (blue), Sheet (red), and Coil (magenta)—and secondary structures composition percentages (bottom panel).
