# Supplementary material for: Generation, characterization, and application of caprine herpesvirus 1 secreted glycoprotein D
Source: Microbiol Spectr. 2025 Nov 28;14(1):e02373-25. doi: 10.1128/spectrum.02373-25 (PMC12772238; doi:10.1128/spectrum.02373-25)
Supplement: File S3 — Top 10 of the 172 templates matching the CpHV-1 gD sequence on SWISS-MODEL. [file spectrum.02373-25-s0003.docx]

| **Name** | **Seq Identity** | **Seq Sim** | **Coverage** | **Resolution** | **Method** | **GMQE** | **Found By** |
| --- | --- | --- | --- | --- | --- | --- | --- |
| 6lsa.2.B | 70.270 | 0.532 | 0.545 | 2.204 | X-ray | 0.498 | BLAST |
| 6lsa.1.B | 70.270 | 0.532 | 0.545 | 2.204 | X-ray | 0.495 | BLAST |
| 6ls9.2.A | 70.270 | 0.532 | 0.545 | 2.503 | X-ray | 0.493 | BLAST |
| 6ls9.3.A | 70.270 | 0.532 | 0.545 | 2.503 | X-ray | 0.488 | BLAST |
| 6lsa.2.B | 65.108 | 0.509 | 0.683 | 2.204 | X-ray | 0.573 | HHblits |
| 6lsa.1.B | 65.108 | 0.509 | 0.683 | 2.204 | X-ray | 0.572 | HHblits |
| 6ls9.2.A | 65.108 | 0.509 | 0.683 | 2.503 | X-ray | 0.565 | HHblits |
| 6ls9.3.A | 65.108 | 0.509 | 0.683 | 2.503 | X-ray | 0.561 | HHblits |
| 5x5w.1.A | 43.318 | 0.42 | 0.533 | 2.700 | X-ray | 0.4 | BLAST |
| 5x5v.2.A | 43.318 | 0.42 | 0.533 | 1.500 | X-ray | 0.398 | BLAST |

**Supplementary file 3:** Top 10 of the 172 templates matching the CpHV-1 gD sequence on SWISS-MODEL. Templates were ranked based on sequence identity. Each entry includes sequence identity, similarity, coverage, resolution, method of structure determination, GMQE and database.
