# Supplementary material for: Generation, characterization, and application of caprine herpesvirus 1 secreted glycoprotein D
Source: Microbiol Spectr. 2025 Nov 28;14(1):e02373-25. doi: 10.1128/spectrum.02373-25 (PMC12772238; doi:10.1128/spectrum.02373-25)
Supplement: File S4 — Structural assessment of CpHV-1 gD models. [file spectrum.02373-25-s0004.docx]

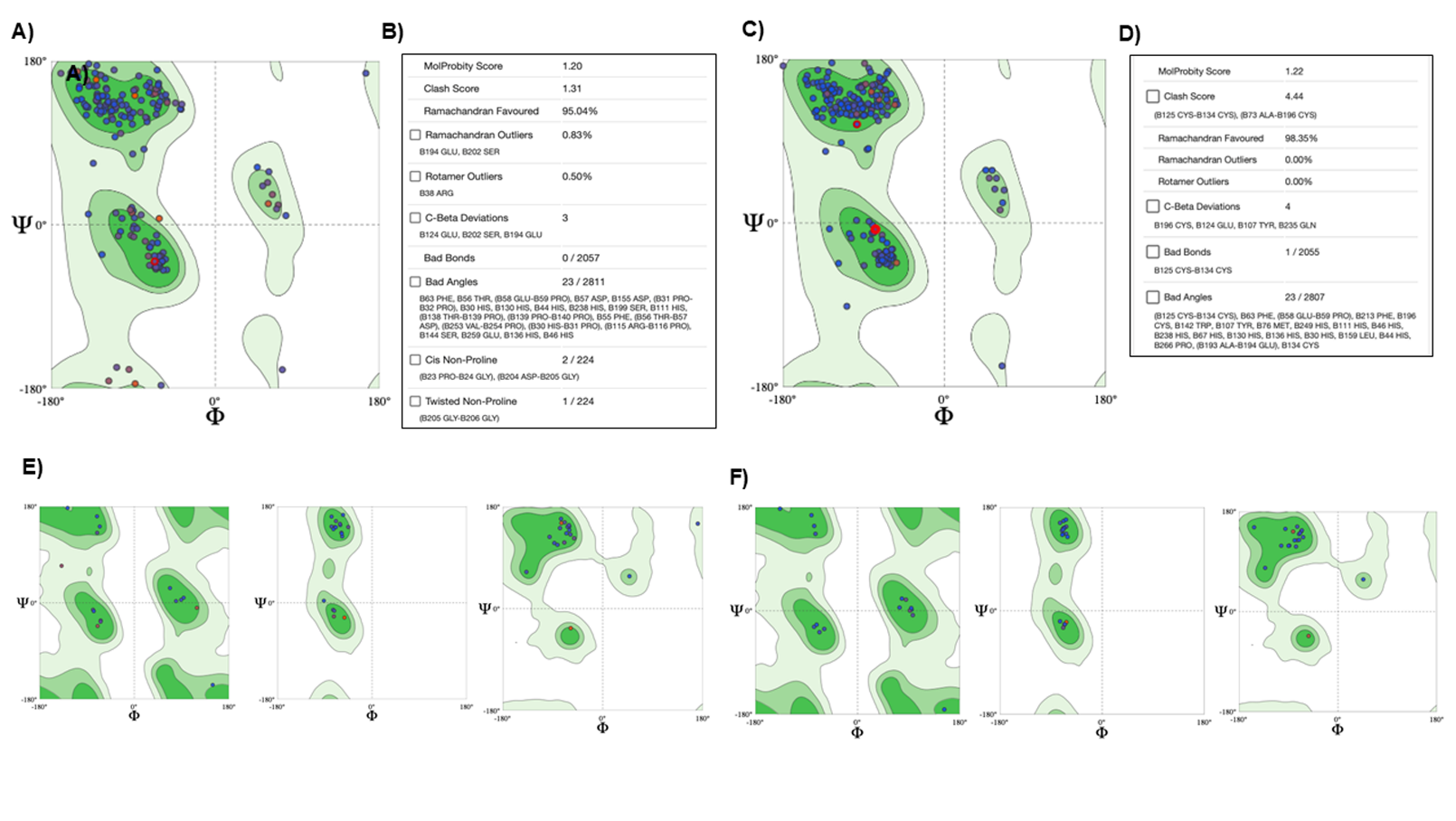


**Supplementary File 4.**  **Structural assessment of CpHV-1 gD models** showing improved structure correctness of the GalaxyRefine model. Ramachandran plots of the crude **(A)** and refined models **(B)** generated with SWISS-MODEL confirming protein structure based on energetically allowed and disallowed dihedral angles psi (ψ) and phi (φ) of amino acid residues. Structure quality reports of crude **(B)** and refined models **(D).** Ramachandran plots for specific residue types (from left to right - glycine, pre-proline, and proline) of crude **(E)** and refined CpHV-1 gD **(F)**.
