# Supplementary material for: Generation, characterization, and application of caprine herpesvirus 1 secreted glycoprotein D
Source: Microbiol Spectr. 2025 Nov 28;14(1):e02373-25. doi: 10.1128/spectrum.02373-25 (PMC12772238; doi:10.1128/spectrum.02373-25)
Supplement: File S5 — Sec-gD mutants' generation. [file spectrum.02373-25-s0005.docx]

MUT1

atgtgggccctcgtgctcgcagcgctaagcgcgctcggggcgctgctggccgcgccgacgtccgagcccgggacgaccgtgtatgtgcatccgccgacatacccgccgccgcggtaccaatacacggagcactggcacgtcaacgccccggtcccgtcgccgtttaccgacgagcccgcgcggcgctttgaggtgcgccacgtaacgagcggctcggcgtgcggcatgctggccctcatcgcggatgcccaggtggggcggaccctgtggggggtggcgcgccggcaggggcgcacgtacaacgccacggtcgcgtggtaccggatagagcacggctgcgcccggccgctgtacgtgatggagtaccaagagtgcgaccccaataagcactttggctactgccggcaccgcacccctccgttttgggccagctttctgtctgggttcgcgtacaccacggcggatgagctggggctcgtcatggccgcgcccgcgaagctcgtcgagggccagtaccggcgggccgtgtacatcgacaacaaggccacctacaccgacttcatggtctcgctgcccgcagagagctgctggttctctaggcgcagcactgacggcgggtacaccttcagcgcctgcttcgcggcctcggactacgaacaggggcgcgtgcagcggatggcgtacctcctccagtactacccccaagaggcgcacaaggccatggtggattactggtacatgagccacgggggcgttgtgcccccgtacttcgaggaggcgacgcgctacgagcgcccgccagcgccccccagccgcgtcacccccacgcccaatggcccggggggcggcgaagacggcgagggggccgccgacggagacccggaggcaagccgccccgcggaagaggcggacggcgagacccccggtcgcgggccagagagcgaaggcgaacacgccccgggcggccgcgccgacgcgagccggcccgaaggctggccgagcctcgaagacatcacgcgggcgccggacccgcctacgacgcctaccctcccgcccgccgcgccgtacccctacgacgtgcccgattacgcctaa

MWALVLAALSALGALLAAPTSEPGTTVYVHPPTYPPPRYQYTEHWHVNAPVPSPFTDEPARRFEVRHVTSGSACGMLALIADAQVGRTLWGVARRQGRTYNATVAWYRIEHGCARPLYVMEYQECDPNKHFGYCRHRTPPFWASFLSGFAYTTADELGLVMAAPAKLVEGQYRRAVYIDNKATYTDFMVSLPAESCWFSRRSTDGGYTFSACFAASDYEQGRVQRMAYLLQYYPQEAHKAMVDYWYMSHGGVVPPYFEEATRYERPPAPPSRVTPTPNGPGGGEDGEGAADGDPEASRPAEEADGETPGRGPESEGEHAPGGRADASRPEGWPSLEDITRAPDPPTTPTLPPAAPYPYDVPDYA-

**Name: MUT1 Length: 364**

**MWALVLAALSALGALLAAPTSEPGTTVYVHPPTYPPPRYQYTEHWHVNAPVPSPFTDEPARRFEVRHVTSGSACGMLALI 80**

**ADAQVGRTLWGVARRQGRTYNATVAWYRIEHGCARPLYVMEYQECDPNKHFGYCRHRTPPFWASFLSGFAYTTADELGLV 160**

**MAAPAKLVEGQYRRAVYIDNKATYTDFMVSLPAESCWFSRRSTDGGYTFSACFAASDYEQGRVQRMAYLLQYYPQEAHKA 240**

**MVDYWYMSHGGVVPPYFEEATRYERPPAPPSRVTPTPNGPGGGEDGEGAADGDPEASRPAEEADGETPGRGPESEGEHAP 320**

**GGRADASRPEGWPSLEDITRAPDPPTTPTLPPAAPYPYDVPDYA 400**

**................................................................................ 80**

**....................N........................................................... 160**

**................................................................................ 240**

**................................................................................ 320**

**............................................ 400**

**(Threshold=0.5)**

**----------------------------------------------------------------------**

**SeqName Position Potential Jury N-Glyc**

**agreement result**

**----------------------------------------------------------------------**

**Sequence 101 NATV 0.6543 (7/9) +**

**----------------------------------------------------------------------**

**
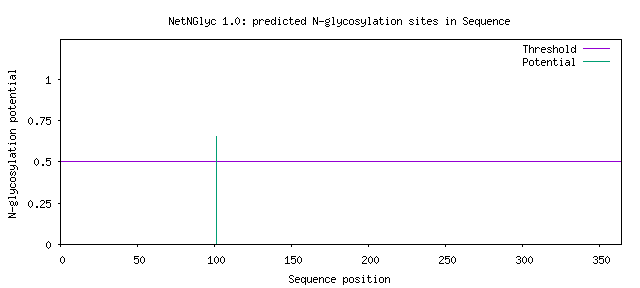
**

MUT2

atgtgggccctcgtgctcgcagcgctaagcgcgctcggggcgctgctggccgcgccgacgtccgagcccgggacgaccgtgtatgtgcatccgccgacatacccgccgccgcggtacaactacacggagcactggcacgtcaacgccccggtcccgtcgccgtttaccgacgagcccgcgcggcgctttgaggtgcgccacgtaacgagcggctcggcgtgcggcatgctggccctcatcgcggatgcccaggtggggcggaccctgtggggggtggcgcgccggcaggggcgcacgtaccaagccacggtcgcgtggtaccggatagagcacggctgcgcccggccgctgtacgtgatggagtaccaagagtgcgaccccaataagcactttggctactgccggcaccgcacccctccgttttgggccagctttctgtctgggttcgcgtacaccacggcggatgagctggggctcgtcatggccgcgcccgcgaagctcgtcgagggccagtaccggcgggccgtgtacatcgacaacaaggccacctacaccgacttcatggtctcgctgcccgcagagagctgctggttctctaggcgcagcactgacggcgggtacaccttcagcgcctgcttcgcggcctcggactacgaacaggggcgcgtgcagcggatggcgtacctcctccagtactacccccaagaggcgcacaaggccatggtggattactggtacatgagccacgggggcgttgtgcccccgtacttcgaggaggcgacgcgctacgagcgcccgccagcgccccccagccgcgtcacccccacgcccaatggcccggggggcggcgaagacggcgagggggccgccgacggagacccggaggcaagccgccccgcggaagaggcggacggcgagacccccggtcgcgggccagagagcgaaggcgaacacgccccgggcggccgcgccgacgcgagccggcccgaaggctggccgagcctcgaagacatcacgcgggcgccggacccgcctacgacgcctaccctcccgcccgccgcgccgtacccctacgacgtgcccgattacgcctaa

MWALVLAALSALGALLAAPTSEPGTTVYVHPPTYPPPRYNYTEHWHVNAPVPSPFTDEPARRFEVRHVTSGSACGMLALIADAQVGRTLWGVARRQGRTYQATVAWYRIEHGCARPLYVMEYQECDPNKHFGYCRHRTPPFWASFLSGFAYTTADELGLVMAAPAKLVEGQYRRAVYIDNKATYTDFMVSLPAESCWFSRRSTDGGYTFSACFAASDYEQGRVQRMAYLLQYYPQEAHKAMVDYWYMSHGGVVPPYFEEATRYERPPAPPSRVTPTPNGPGGGEDGEGAADGDPEASRPAEEADGETPGRGPESEGEHAPGGRADASRPEGWPSLEDITRAPDPPTTPTLPPAAPYPYDVPDYA-

**Name: MUT2 Length: 364**

**MWALVLAALSALGALLAAPTSEPGTTVYVHPPTYPPPRYNYTEHWHVNAPVPSPFTDEPARRFEVRHVTSGSACGMLALI 80**

**ADAQVGRTLWGVARRQGRTYQATVAWYRIEHGCARPLYVMEYQECDPNKHFGYCRHRTPPFWASFLSGFAYTTADELGLV 160**

**MAAPAKLVEGQYRRAVYIDNKATYTDFMVSLPAESCWFSRRSTDGGYTFSACFAASDYEQGRVQRMAYLLQYYPQEAHKA 240**

**MVDYWYMSHGGVVPPYFEEATRYERPPAPPSRVTPTPNGPGGGEDGEGAADGDPEASRPAEEADGETPGRGPESEGEHAP 320**

**GGRADASRPEGWPSLEDITRAPDPPTTPTLPPAAPYPYDVPDYA 400**

**.......................................N........................................ 80**

**................................................................................ 160**

**................................................................................ 240**

**................................................................................ 320**

**............................................ 400**

**(Threshold=0.5)**

**----------------------------------------------------------------------**

**SeqName Position Potential Jury N-Glyc**

**agreement result**

**----------------------------------------------------------------------**

**Sequence 40 NYTE 0.7796 (9/9) +++**

**----------------------------------------------------------------------**

**
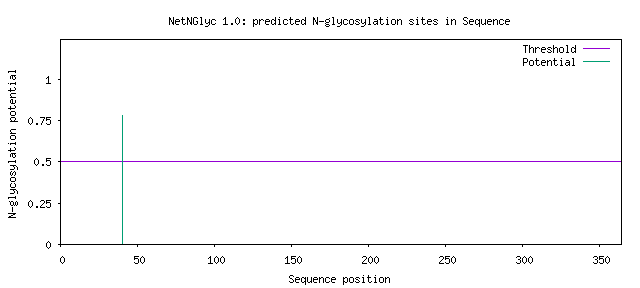
**

**Site 1 mutation**

ccg ccg ccg cgg tac aac tac acg gag cac tgg Unmutated
 P   P   P   R   Y   N   Y   T   E   H   W

ccg ccg ccg cgg tac caa tac acg gag cac tgg Mutated
 P   P   P   R   Y   Q   Y   T   E   H   W

Mut1-Sense 5’-CCG CCG CCG CGG TAC CAA TAC ACG GAG CAC TGG-3’

Mut1-Anti 5’- CCA GTG CTC CGT GTA TTG GTA CCG CGG CGG CGG-3’

**Site 2 mutation**

cag ggg cgc acg tac aac gcc acg gtc gcg tgg Unmutated
 Q   G   R   T   Y   N   A   T   V   A   W

cag ggg cgc acg tac caa gcc acg gtc gcg tgg Mutated
 Q   G   R   T   Y   Q   A   T   V   A   W

Mut2-Sense 5’- CAG GGG CGC ACG TAC CAA GCC ACG GTC GCG TGG-3’

Mut2-Anti 5’- CCA CGC GAC CGT GGC TTG GTA CGT GCG CCC CTG-5’

**Supplementary File 5. Sec-gD mutants’ generation.** Nucleotides and sec-gD amino acid sequences with mutated N-linked glycosylation sites (MUT1 and MUT2). In these sequences, Asparagine (N) residues were substituted with Glutamine (Q), highlighted in green. According to predictions from NetGlyc 1.0, potential glycosylation sites are marked in yellow, while mutated sites (N → Q) are marked in green. A score above the default threshold of 0.5 indicates a predicted glycosylation site, provided it occurs within the required motif Asn-Xaa-Ser/Thr (where Xaa ≠ Proline). The “potential” score represents the average output from nine neural networks. For additional context, the jury agreement column shows how many of these nine networks support the prediction.

Primers labeled Site 1 mutation and Site 2 mutation were used for site-directed mutagenesis.
