# Supplementary material for: Generation, characterization, and application of caprine herpesvirus 1 secreted glycoprotein D
Source: Microbiol Spectr. 2025 Nov 28;14(1):e02373-25. doi: 10.1128/spectrum.02373-25 (PMC12772238; doi:10.1128/spectrum.02373-25)
Supplement: File S6 — ROC curve coordinates. [file spectrum.02373-25-s0006.docx]

ROC curve coordinates

| **Serum dilution** | **Positive if greater than or equal to** | **Sensitivity** | **1 - Specificity** | **Youden index** |
| --- | --- | --- | --- | --- |
| **1:10** | -0,867100 | 1,000 | 1,000 | 0,000 |
|  | 0,145400 | 1,000 | 0,900 | 0,100 |
|  | 0,185250 | 1,000 | 0,800 | 0,200 |
|  | 0,240450 | 1,000 | 0,700 | 0,300 |
|  | 0,276250 | 1,000 | 0,600 | 0,400 |
|  | 0,286450 | 1,000 | 0,500 | 0,500 |
|  | 0,303600 | 1,000 | 0,400 | 0,600 |
|  | 0,322250 | 1,000 | 0,300 | 0,700 |
|  | 0,346350 | 1,000 | 0,200 | 0,800 |
|  | 0,417750 | 1,000 | 0,100 | 0,900 |
|  | 0,475200 | 0,900 | 0,100 | 0,800 |
|  | 0,546950 | 0,900 | 0,000 | 0,900 |
|  | 0,661100 | 0,800 | 0,000 | 0,800 |
|  | 0,764350 | 0,700 | 0,000 | 0,700 |
|  | 0,892950 | 0,600 | 0,000 | 0,600 |
|  | 1,061800 | 0,500 | 0,000 | 0,500 |
|  | 1,197750 | 0,400 | 0,000 | 0,400 |
|  | 1,252750 | 0,300 | 0,000 | 0,300 |
|  | 1,299100 | 0,200 | 0,000 | 0,200 |
|  | 1,449500 | 0,100 | 0,000 | 0,100 |
|  | 2,567300 | 0,000 | 0,000 | 0,000 |
| **1:20** | -0,833800 | 1,000 | 1,000 | 0,000 |
|  | 0,168800 | 1,000 | 0,900 | 0,100 |
|  | 0,171800 | 1,000 | 0,800 | 0,200 |
|  | 0,177850 | 1,000 | 0,700 | 0,300 |
|  | 0,186450 | 1,000 | 0,600 | 0,400 |
|  | 0,191750 | 1,000 | 0,500 | 0,500 |
|  | 0,197250 | 1,000 | 0,400 | 0,600 |
|  | 0,205200 | 1,000 | 0,300 | 0,700 |
|  | 0,245000 | 1,000 | 0,200 | 0,800 |
|  | 0,280550 | 0,900 | 0,200 | 0,700 |
|  | 0,281300 | 0,900 | 0,100 | 0,800 |
|  | 0,316900 | 0,900 | 0,000 | 0,900 |
|  | 0,367200 | 0,800 | 0,000 | 0,800 |
|  | 0,473800 | 0,700 | 0,000 | 0,700 |
|  | 0,592500 | 0,600 | 0,000 | 0,600 |
|  | 0,745700 | 0,500 | 0,000 | 0,500 |
|  | 0,875600 | 0,400 | 0,000 | 0,400 |
|  | 0,914750 | 0,300 | 0,000 | 0,300 |
|  | 0,993400 | 0,200 | 0,000 | 0,200 |
|  | 1,162150 | 0,100 | 0,000 | 0,100 |
|  | 2,287700 | 0,000 | 0,000 | 0,000 |
|  | 0,000000 | 1,000 | 1,000 | 0,000 |

| **1:40** | 0,111600 | 1,000 | 0,900 | 0,100 |
| --- | --- | --- | --- | --- |
|  | 0,121550 | 1,000 | 0,800 | 0,200 |
|  | 0,132100 | 1,000 | 0,700 | 0,300 |
|  | 0,141050 | 1,000 | 0,600 | 0,400 |
|  | 0,150300 | 1,000 | 0,500 | 0,500 |
|  | 0,153950 | 1,000 | 0,400 | 0,600 |
|  | 0,171100 | 1,000 | 0,300 | 0,700 |
|  | 0,186250 | 1,000 | 0,200 | 0,800 |
|  | 0,190350 | 1,000 | 0,100 | 0,900 |
|  | 0,199100 | 1,000 | 0,000 | 1,000 |
|  | 0,221150 | 0,900 | 0,000 | 0,900 |
|  | 0,251100 | 0,800 | 0,000 | 0,800 |
|  | 0,277300 | 0,700 | 0,000 | 0,700 |
|  | 0,350700 | 0,600 | 0,000 | 0,600 |
|  | 0,427500 | 0,500 | 0,000 | 0,500 |
|  | 0,543950 | 0,400 | 0,000 | 0,400 |
|  | 0,690000 | 0,300 | 0,000 | 0,300 |
|  | 0,748200 | 0,200 | 0,000 | 0,200 |
|  | 0,792450 | 0,100 | 0,000 | 0,100 |
|  | 1,000000 | 0,000 | 0,000 | 0,000 |
| **1:80** | 0,000000 | 1,000 | 1,000 | 0,000 |
|  | 0,087800 | 1,000 | 0,900 | 0,100 |
|  | 0,095900 | 1,000 | 0,800 | 0,200 |
|  | 0,098000 | 1,000 | 0,700 | 0,300 |
|  | 0,099350 | 1,000 | 0,600 | 0,400 |
|  | 0,100650 | 1,000 | 0,500 | 0,500 |
|  | 0,101850 | 1,000 | 0,400 | 0,600 |
|  | 0,104400 | 1,000 | 0,300 | 0,700 |
|  | 0,113950 | 1,000 | 0,200 | 0,800 |
|  | 0,124050 | 1,000 | 0,100 | 0,900 |
|  | 0,126950 | 1,000 | 0,000 | 1,000 |
|  | 0,139550 | 0,900 | 0,000 | 0,900 |
|  | 0,153750 | 0,800 | 0,000 | 0,800 |
|  | 0,178750 | 0,700 | 0,000 | 0,700 |
|  | 0,221100 | 0,600 | 0,000 | 0,600 |
|  | 0,272150 | 0,500 | 0,000 | 0,500 |
|  | 0,311000 | 0,400 | 0,000 | 0,400 |
|  | 0,374850 | 0,300 | 0,000 | 0,300 |
|  | 0,460100 | 0,200 | 0,000 | 0,200 |
|  | 0,537200 | 0,100 | 0,000 | 0,100 |
|  | 1,000000 | 0,000 | 0,000 | 0,000 |
|  | 0,000000 | 1,000 | 1,000 | 0,000 |
|  | 0,065200 | 1,000 | 0,900 | 0,100 |
|  | 0,069350 | 1,000 | 0,800 | 0,200 |
|  | 0,074300 | 1,000 | 0,700 | 0,300 |
|  | 0,077800 | 1,000 | 0,600 | 0,400 |
|  | 0,083250 | 1,000 | 0,500 | 0,500 |
|  | 0,087150 | 1,000 | 0,400 | 0,600 |

| **1:160** | 0,090200 | 1,000 | 0,300 | 0,700 |
| --- | --- | --- | --- | --- |
|  | 0,097600 | 1,000 | 0,200 | 0,800 |
|  | 0,104100 | 0,900 | 0,200 | 0,700 |
|  | 0,108600 | 0,900 | 0,100 | 0,800 |
|  | 0,112800 | 0,800 | 0,100 | 0,700 |
|  | 0,115600 | 0,800 | 0,000 | 0,800 |
|  | 0,128250 | 0,700 | 0,000 | 0,700 |
|  | 0,147250 | 0,600 | 0,000 | 0,600 |
|  | 0,165900 | 0,500 | 0,000 | 0,500 |
|  | 0,177150 | 0,400 | 0,000 | 0,400 |
|  | 0,239750 | 0,300 | 0,000 | 0,300 |
|  | 0,315900 | 0,200 | 0,000 | 0,200 |
|  | 0,388450 | 0,100 | 0,000 | 0,100 |
|  | 1,000000 | 0,000 | 0,000 | 0,000 |
| **1:320** | 0,000000 | 1,000 | 1,000 | 0,000 |
|  | 0,061900 | 1,000 | 0,900 | 0,100 |
|  | 0,062350 | 1,000 | 0,800 | 0,200 |
|  | 0,063050 | 1,000 | 0,700 | 0,300 |
|  | 0,064300 | 1,000 | 0,600 | 0,400 |
|  | 0,066800 | 1,000 | 0,500 | 0,500 |
|  | 0,068800 | 1,000 | 0,400 | 0,600 |
|  | 0,069650 | 1,000 | 0,300 | 0,700 |
|  | 0,070800 | 1,000 | 0,200 | 0,800 |
|  | 0,071300 | 0,900 | 0,100 | 0,800 |
|  | 0,074100 | 0,800 | 0,100 | 0,700 |
|  | 0,080150 | 0,800 | 0,000 | 0,800 |
|  | 0,088000 | 0,700 | 0,000 | 0,700 |
|  | 0,100400 | 0,600 | 0,000 | 0,600 |
|  | 0,109200 | 0,500 | 0,000 | 0,500 |
|  | 0,114600 | 0,400 | 0,000 | 0,400 |
|  | 0,152150 | 0,300 | 0,000 | 0,300 |
|  | 0,198250 | 0,200 | 0,000 | 0,200 |
|  | 0,257100 | 0,100 | 0,000 | 0,100 |
|  | 1,000000 | 0,000 | 0,000 | 0,000 |
| **1:640** | 0,000000 | 1,000 | 1,000 | 0,000 |
|  | 0,056750 | 1,000 | 0,900 | 0,100 |
|  | 0,057700 | 1,000 | 0,800 | 0,200 |
|  | 0,058700 | 0,900 | 0,700 | 0,200 |
|  | 0,060700 | 0,900 | 0,600 | 0,300 |
|  | 0,062900 | 0,900 | 0,500 | 0,400 |
|  | 0,064000 | 0,800 | 0,500 | 0,300 |
|  | 0,065000 | 0,800 | 0,400 | 0,400 |
|  | 0,065300 | 0,800 | 0,300 | 0,500 |
|  | 0,066900 | 0,700 | 0,300 | 0,400 |
|  | 0,069200 | 0,700 | 0,200 | 0,500 |
|  | 0,072650 | 0,700 | 0,100 | 0,600 |
|  | 0,076500 | 0,700 | 0,000 | 0,700 |
|  | 0,079200 | 0,600 | 0,000 | 0,600 |

|  | 0,082650 | 0,500 | 0,000 | 0,500 |
| --- | --- | --- | --- | --- |
|  | 0,085200 | 0,400 | 0,000 | 0,400 |
|  | 0,101400 | 0,300 | 0,000 | 0,300 |
|  | 0,155550 | 0,200 | 0,000 | 0,200 |
|  | 0,210250 | 0,100 | 0,000 | 0,100 |
|  | 1,000000 | 0,000 | 0,000 | 0,000 |
| **1:1280** | 0,000000 | 1,000 | 1,000 | 0,000 |
|  | 0,054600 | 1,000 | 0,900 | 0,100 |
|  | 0,056350 | 1,000 | 0,800 | 0,200 |
|  | 0,057500 | 1,000 | 0,700 | 0,300 |
|  | 0,058150 | 1,000 | 0,600 | 0,400 |
|  | 0,059100 | 1,000 | 0,500 | 0,500 |
|  | 0,060100 | 1,000 | 0,400 | 0,600 |
|  | 0,060850 | 0,900 | 0,400 | 0,500 |
|  | 0,061650 | 0,900 | 0,300 | 0,600 |
|  | 0,064100 | 0,900 | 0,200 | 0,700 |
|  | 0,066550 | 0,800 | 0,200 | 0,600 |
|  | 0,067500 | 0,800 | 0,100 | 0,700 |
|  | 0,068650 | 0,700 | 0,100 | 0,600 |
|  | 0,069150 | 0,600 | 0,100 | 0,500 |
|  | 0,071600 | 0,500 | 0,100 | 0,400 |
|  | 0,075900 | 0,500 | 0,000 | 0,500 |
|  | 0,083450 | 0,400 | 0,000 | 0,400 |
|  | 0,106400 | 0,300 | 0,000 | 0,300 |
|  | 0,132600 | 0,200 | 0,000 | 0,200 |
|  | 0,148100 | 0,100 | 0,000 | 0,100 |
|  | 1,000000 | 0,000 | 0,000 | 0,000 |
